# The Pocket Anatomist

**Published:** 1894-12

**Authors:** 


THE POCKET ANATOMIST. By C. Henri Leonard, A. M., M. D., Professor of Gynecology, Detroit College of Medicine. Leather, 300 pages, 193 illustrations, post-paid, $1.00. The Illustrated Medical Journal Co., Publishers, Detroit, Mich,

This is quite a valuable addition to the library or desk of any physician or student. The text and illustrations are founded on Gray and are, of course, correct. The most valuable feature of this little volume is the "Dissection Hints and Visceral Anatomy," which will prove of great assistance to students of medicine in assisting them in their dissections.

It is printed on good paper and the press work and illustrations are both much better than one would expect considering the extremely low price at which it is offered. G. B.

